# Quantifying the impact of community quarantine on SARS transmission in Ontario: estimation of secondary case count difference and number needed to quarantine

**DOI:** 10.1186/1471-2458-9-488

**Published:** 2009-12-24

**Authors:** Susan J Bondy, Margaret L Russell, Julie ML Laflèche, Elizabeth Rea

**Affiliations:** 1 Dalla Lana School of Public Health, University of Toronto, 155 College Street, 6th Floor, Toronto, ON, MT5 3M7, Canada; 2 Department of Community Health Sciences, University of Calgary, 3280 Hospital Dr NW, Calgary, Alberta, T2N 4Z6, Canada; 3 Toronto Public Health, 277 Victoria St, Toronto, Ontario, M5B 1W2, Canada

## Abstract

**Background:**

Community quarantine is controversial, and the decision to use and prepare for it should be informed by specific quantitative evidence of benefit. Case-study reports on 2002-2004 SARS outbreaks have discussed the role of quarantine in the community in transmission. However, this literature has not yielded quantitative estimates of the reduction in secondary cases attributable to quarantine as would be seen in other areas of health policy and cost-effectiveness analysis.

**Methods:**

Using data from the 2003 Ontario, Canada, SARS outbreak, two novel expressions for the impact of quarantine are presented. Secondary Case Count Difference (SCCD) reflects reduction in the average number of transmissions arising from a SARS case in quarantine, relative to not in quarantine, at onset of symptoms. SCCD was estimated using Poisson and negative binomial regression models (with identity link function) comparing the number of secondary cases to each index case for quarantine relative to non-quarantined index cases. The inverse of this statistic is proposed as the number needed to quarantine (NNQ) to prevent one additional secondary transmission.

**Results:**

Our estimated SCCD was 0.133 fewer secondary cases per quarantined versus non-quarantined index case; and a NNQ of 7.5 exposed individuals to be placed in community quarantine to prevent one additional case of transmission in the community. This analysis suggests quarantine can be an effective preventive measure, although these estimates lack statistical precision.

**Conclusions:**

Relative to other health policy areas, literature on quarantine tends to lack in quantitative expressions of effectiveness, or agreement on how best to report differences in outcomes attributable to control measure. We hope to further this discussion through presentation of means to calculate and express the impact of population control measures. The study of quarantine effectiveness presents several methodological and statistical challenges. Further research and discussion are needed to understand the costs and benefits of enacting quarantine, and this includes a discussion of how quantitative benefit should be communicated to decision-makers and the public, and evaluated.

## Background

Outbreaks such as Severe Acute Respiratory Syndrome (SARS) and H1N1 influenza have triggered acute policy debates around community-based infection control measures [1-4]. Quarantine is the segregation of healthy persons exposed to an infectious disease from unexposed persons, until the exposed person becomes ill or the incubation period has passed [2,5]. Quarantine is also one of the most controversial measures available in outbreak control, with important impacts on economic activity and civil liberties [6,7]. Public health agencies need to have weighed evidence of the potential costs and benefits of these manoeuvres before the next outbreak of a severe, novel infection [8,9] and to have publicly and widely communicated the importance of planned control measures [6,10]. This makes outcome evaluation of modern quarantine activity an important area for research [2,4,6].

Health policy and practice guidelines rely heavily on experimental and quasi-experimental research comparing outcomes under intervention and non-intervention conditions [11-14]. Statistical estimates used in health decision-making tend to derive from differences in outcome rates in treated versus non-treated individuals (e.g., risk difference, RD; attributable risk, AR and population attributable risk, PAR) [12,13,15], as well as variations on the number needed to treat (NNT) statistic [11,15-20]. Research to evaluate the impact of modern quarantine is less well-developed, and no clear tradition has emerged in the measures of association used to communicate net harm and benefit. The quantitative impact of control measures for infectious diseases is commonly assessed using variants of the effective reproductive number, R (the average observed number of secondary infections per index cases in an observed population). R_t _the daily effective reproductive number, is the observed number of secondary cases to index case at a specific time. When R_t, _has a value below1.0, this is evidence that an outbreak is dying out [21].

Here, we discuss the quantitative evidence available to inform policy-makers on the potential benefit of community-based quarantine in outbreak control, based upon the SARS experience. To bridge the gap between evaluative research on quarantine and other areas, we also use data from the 2003 Ontario, Canada (hereafter, Ontario), SARS outbreak to demonstrate statistical approaches used elsewhere to the evaluation of modern quarantine measures.

We propose a series of measures of reduction of adverse outcomes (analogous to those used in other contexts), to evaluation of quarantine. Specifically, we estimated the difference in secondary transmissions that is attributable to community quarantine as the Secondary Case Count Difference (SCCD), which is comparable to risk difference statistics and interpretable as reduction in average transmissions per existing case, attributable to intervention. We also report the inverse of this statistic, interpretable as Number Need to Quarantine (NNQ).

## Methods

### Subjects and data extraction

The contact tracing and quarantine procedures used in the 2003 Ontario SARS outbreak have been described previously [22,23]. From source public health records, we extracted data for all 332 index cases with a final disposition of suspect or probable SARS [23] of whom 204 had at least one community contact uniquely associated with them in Public Health records. Contacts included had non-overlapping periods of exposure to SARS index cases within 10 days, as previously reported [23]. The total number of community contacts associated with these index cases, for this analysis, was 8,498. This number excluded health care providers who were contacts of those SARS index cases only as a result of providing care to SARS cases, but included health care workers exposed through social or family contacts. Community contacts were classified by closest level of exposure to the index case (e.g., level 1 being closest at ≥30 minutes within a distance of one metre [23]). For all community contacts, outcome status as a secondary SARS case was defined [23]. We also consider data for an additional 140 individuals who were quarantined as contacts of a known SARS case, became ill and were considered to be potential cases themselves, but who subsequently had SARS ruled out. These additional 140 observations were used only in sensitivity analyses.

### Analysis

In the first step, we carried out an analysis where the 8,498 individual contacts were treated as the unit of analysis. The outcome was whether or not each contact became a SARS case (binary outcome). The predictor variable of interest was whether the associated index case had been in community quarantine at symptom onset. This, seemingly intuitive, analysis treats the individuals in whom the outcome is measured as though they were assigned to treatment conditions. Therefore, information regarding quarantine status is used only from the 204 SARS index cases with one or more community contacts. In place of typical logistic regression which would yield an odds ratio (OR), we obtained risk difference (naïve secondary attack rate difference - ignoring index cases with no contacts) as the measure of association, using a generalized linear regression model with binomial error term and identity link function. Clustering of multiple contacts to index cases was accounted for using robust variance estimates [24,25].

In the next step, we treated all 332 SARS index cases as the unit of analysis and applied Poisson regression analysis to model the number of secondary cases per index case. As before, the exposure of interest is whether or not the index case was quarantined. We calculated several measures of association to describe the difference in transmission observed in the quarantine and non-quarantine group. These included the secondary case count ratio or SSCR, the ratio of secondary cases (per index case) in the quarantine condition relative to the non-quarantine condition. The SSCR is a function of the ratio of secondary cases per quarantined index case (SC_q_) and of the ratio of secondary cases per non-quarantined index case (SC_nq_) as discussed below. We also calculated the difference in average secondary cases per index case between the two groups (secondary case count difference, SCCD), and the inverse of the SCCD which we label as "NNQ".

Secondary Case Count Ratio (SCCR) was estimated using the most familiar form of Poisson regression (Poisson error term and the canonical log link function) [26]. Poisson regression is often used to estimate incidence rate ratios (IRRs), where the outcome variable is the numerator for a rate in a stable population, or where a variable person-time denominator is accounted for by specifying an offset variable for the model. IRR is equal to the exponent of the beta coefficient for the regression term of interest [26,27]. For Poisson models used here, no offset was specified for the number of community contacts at risk for each index case. Said differently, the outcome variable here is not the *rate *of secondary transmission among a variable number of potentially exposed community contacts per index case, but the *number *of secondary SARS cases arising from an index case. The SCCR is the exponentiated beta term for this model (where quarantine status was entered as a binary variable). SCCR is the ratio of (secondary cases per quarantined index case, SC_q_) to (secondary cases per non-quarantined index case, SC_nq_); therefore, SCCR = SC_q_/SC_nq_.

Estimation of SCCD (attributable difference in number of secondary cases, per index case) was achieved using a Poisson model estimating additive effects (Poisson error term and identity link function). SCCD is the difference in the average absolute number of secondary cases in the quarantine versus non-quarantine conditions (SCCD = SC_nq_-SC_q_). This model yields a (non-exponentiated) beta coefficient interpreted as the difference in outcome [26]. Here, beta = SCCD. The inverse of this SCCD we define as NNQ, a statistic analogous to NNT [17,18,28]. NNQ is the number of cases that would need to be in quarantine at symptom onset to prevent one case among contacts of an index case. This is defined as:

Additional analysis quantified the degree to which reduction in total contacts, and close contacts, mediated the impact of quarantine. An estimate of the reduction in size of the SCCR was tested against a null (no change) using a bootstrap method [29]. This mediation analysis was carried out using the canonical (log link) Poisson regression model, above.

For the sensitivity analysis, we re-estimated SCCD and NNQ including data for an additional 140 case observations. This group consisted of quarantined contacts who were, prospectively, under suspicion of being SARS cases themselves (i.e., during the outbreak), but in whom SARS was later excluded. These people also had at least one community contact themselves, in order to be included in the database. This sensitivity analysis was to show the effect of false positive index cases on apparent outcomes of quarantine.

For all regression models, diagnostics were performed. This included post-fit examination for non-linearity for ordinal independent variables (i.e., numbers of contact by level of exposure). Where found, non-linearity was corrected by taking a linear transformation of the predictor variable to obtain normally distributed residuals. Nonnormality of residuals in models including total contacts was corrected by transforming the total number of the contacts (replaced with square root of value) to reduce the influence of the positive skew in this covariate. For all models using the Poisson error term, tests for over-dispersion were carried out. Identical models using the negative binomial error term were also run, correcting for over-dispersion. Both are presented.

Because of the limited sample size, confidence limits based on large sample assumptions are questionable. Therefore, we also present confidence intervals estimated using bootstrap variance estimation [30]. For bootstrapping, 5000 replications were used in all cases. All analyses were carried out with Stata, Version 10 [25].

This study received ethical review and approval from the Health Sciences I Research Ethics Committee of the University of Toronto (Protocol #10764).

## Results

Table 1 summarizes quarantine status for 332 probable and suspect SARS cases in the 2003 Ontario SARS outbreak, with numbers of contacts by level of contact and transmission status.

**Table 1:** Numbers of index SARS cases* and non-overlapping contacts† associated with index cases.

Quarantine status	**N of SARS probable and suspect index cases**†	N of SARS index cases with no community contacts	N of SARS index cases with at least one community contact at any level	Group total numbers of community contacts (and secondary cases) by level of contact‡§				
				**All levels**	**Level 1**	**Level 2A**	**Level 2B**	**Level 3**
				
**Not in quarantine at symptom onset**	267	89	178	7970 (52)	578 (37)	3186 (14)	1258 (1)	2948 (0)
**In quarantine prior to symptom onset**	65	39	26	528 (4)	52 (2)	164 (1)	297 (1)	15 (0)

Total	332	128	204	8498 (56)	630 (39)	3483 (15)	1555 (2)	2963 (0)

*Cases of Severe Acute Respiratory Syndrome (SARS) in the Ontario, Canada, 2003 outbreak. Includes all cases with a final disposition of suspect or probable SARS.

† Also considered, in sensitivity analyses only, were an additional 140 individuals who were potential SARS cases at some point during the outbreak and had at least one community contact, but subsequently did not meet criteria for probable or suspect SARS.

‡ Includes 8,498 community contacts with contact to one SARS case and not a second SARS case within 10 days of exposure to the first within any single 10 day period of exposure.

**§ **Level of contact: 1 = contact for at least 30 minutes within 1 metre; 2A = same room for ≥ 30 minutes; 2B = same room for <30 minutes or same floor, regardless of duration; level 3 = distant contact only - was in same building or large social network.

Table 2 presents several measures of association describing the impact of quarantine using regression models described above. In the first, naïve, regression analysis (Approach A in Table 2) the estimated effect was positive (indicating a harmful effect for quarantine) but not statistically significant.

**Table 2:** Quantitative measures of the impact of quarantine on secondary cases in the community*.

Analysis approach	Measure of association obtained from model	Variance estimation method	Estimated value of measure of association	p-value	95% confidence interval	
					Lower limit	Upper limit
A) Generalized linear model with binomial error and identity link (additive logistic). Unit of analysis is community contacts, clustered within shared 204 index cases. (N = 8498).	Naïve Secondary Attack Rate Difference (incorporates information only for 204 index cases with one or more community contact; see text)	Asymptotic: *Bootstrapped:*	0.00105 0.00105†	0.786 *0.881*	-0.00665 *-0.01269*	0.00866 *0.01479*

B) Canonical Poisson and negative binomial regression with log link functions. Unit of analysis is all index cases. (N = 332).	Beta coefficient	Poisson, asymptotic: Neg.Binomial asymptotic: Poisson, *bootstrapped:*	-1.15209 -1.15209 -1.15209†	0.026 0.057 *0.589*	-2.16906 -2.33953 *-5.32984*	-0.13511 0.03535 *3.02567*
	Secondary Case Count Ratio (SCCR) (SCCR = exponentiated Beta for quarantine)	Poisson, asymptotic: Neg.Binomial asymptotic: Poisson, *bootstrapped*:	0.31598 0.31598 0.31598†	0.026 0.057 *0.573*	0.11428 0.09637 *0.00577*	0.87362 1.03599 *17.30158*

C) Generalized linear model with Poisson error term and identity link function, yielding an additive effect measure.	Secondary Case Count Difference (SCCD).(SCCD = Beta coefficient for quarantine)	Poisson, asymptotic: Neg.Binomial, asymptotic Poisson, *bootstrapped*:	-0.13322 -0.13322 -0.13322†	0.001 0.002 *1.000*	-0.21346 -0.21812 *-1.18 × 10^14^*	-0.05298 -0.04832 *1.18 × 10^14^*
Unit of analysis is all index cases. (N = 332).	Number needed to quarantine (NNQ). (NNQ = 1/|SCCD|, above)	Poisson, asymptotic: Neg.Binomial, asymptotic: Poisson, *bootstrapped:*	7.50647 7.50648 7.50647†	0.001 0.002 *1.000*	4.68469 4.58462 *-8.476 × 10^-15^*	18.87661 20.69733 *8.476 × 10^-15^*

* Data from 332 index community-living probable or suspect Severe Acute Respiratory Syndrome (SARS) cases and 8,498 associated community contacts. Ontario, Canada, SARS outbreak, 2003.

† Bootstrapping used to obtain variance estimate; point estimate fixed from corresponding asymptotic model.

The first model treating index cases as the unit of analysis (Approach B in Table 2) yields an estimate of SCCR of 0.316 indicating less than one third the number of secondary cases for quarantined versus non-quarantined index cases. Approach C provides the estimated SCCD obtained using an additive Poisson regression model (Poisson error term and identity link function). The average difference in secondary SARS cases in moving from non-quarantine to quarantine status is estimated at 0.133 secondary cases per index case. The final estimate in Table 2 is NNQ suggesting 7.51 SARS index cases be placed under quarantine to reduce the number of secondary cases by one. When variance estimates and confidence intervals are estimated using a large-sample assumption (asymptotic variance) all Poisson regression models presented were statistically significant. When negative binomial regression was used to estimate SCCR, this became non-significant (p = 0.057). When using bootstrap variance methods, confidence intervals for all estimates became very wide and none of these estimates were significantly different from the null value.

Table 3 presents supplemental analyses of the degree to which the effect of quarantine is explained by reduction in the number of contacts. Adjustment of the effect of quarantine only for the total number of contacts had little impact on the point estimate for SCCR (0.35245 versus 0.31598; data not shown), nor its significance (when compared to Table 2). However, when quarantine status was also adjusted (see Table 3) for the number of close contacts (level 1; see Table 1), the SCCR attributable to quarantine was reduced by 3.6% and it's asymptotic level of significance went from p = 0.026 to p = 0.046. This change falls just short of satisfying criteria for significant mediation using the Baron and Kenny method [31]. A bootstrapped test for reduction of effect [29] was not significant (p > 0.999).

**Table 3:** Assessment of whether number and level of contacts mediate the effect of quarantine on secondary cases.

Term in adjusted model* (contrasts with crude model B, in Table 2)	Secondary Case Count Ratio (SCCR)	p-value under large sample assumption and using bootstrapped variance estimate (in italics)	95% confidence interval under large sample assumption and using bootstrapped variance estimate (in italics)	
			
			Lower limit	Upper limit
Quarantine (v. no)	0.3524498	0.046 *0.550*	0.1266103 *0.0048919*	0.9811279 *20.14694*
Total contacts (continuous†)	0.9999988	0.657 *0.090*	0.9999937 *0.9999816*	1.000004 *1.000017*
Total close contacts (continuous)	1.061969	0.005 *0.009*	1.018544 *1.015351*	1.107246 *1.107246*

* Poisson regression for secondary case count, adjusting for total and close contacts.

†Continuous term transformed by taking the square root of raw data; used to achieve normally-distributed residuals.

The adjusted models in Table 3 also show that the number of close contacts was a significant predictor of the number of secondary cases, even when adjusting for total number of contacts. This remained statistically significant even in the bootstrapped model.

Finally, the SCCD and NNQ estimates (Table 2) were rerun including 140 additional quarantined false-positive SARS cases with no secondary cases. In this analysis, the NNQ estimate drops from 7.51 to 5.74, giving the appearance of a still greater benefit (data not shown; again, statistically significant under the large sample assumption, and not when using bootstrap methods).

For all Poisson-based analyses, model diagnostics did not indicate significant over-dispersion (p-value > 0.99 in all cases). Using negative binomial models to account for over-dispersion did not affect point estimates, but resulted in slightly wider asymptotic confidence limits (Table 2).

## Discussion

### How has quarantine been evaluated?

Previous studies evaluating quarantine for control of SARS used two general methods: simulation studies [9,32,33]; and, case-study reports describing specific settings (e.g. [22,34-42]).

Simulation studies serve several purposes in outbreak research, one of which is to estimate the impact of control measures in outbreak scenarios [1,33]. A review of simulation studies on quarantine effectiveness for SARS has been presented by Bauch and colleagues [9]. This review, and other individual reports [43] suggest that quarantine measures are most effective when mobilized at the very start of the outbreak when numbers are small. While decision-makers must rely heavily on simulation studies, a persistent concern raised [33] is that results are driven by prior assumptions; which may be unrealistic. Simulations rely on some degree of simplification such as considering only point-source outbreaks in non-overlapping populations. More sophisticated models incorporate heterogeneity in parameters including variations in size and behaviour of human contact networks, differences in transmissibility due to the host, and levels of adherence with control measures, such that scenarios simulated reflect what happens in real-world experiences [4,33,44,45]. Simulation studies, typically, do not present impact statistics familiar to health policy makers, and may not provide evidence as compelling to decision-makers as real world outbreak experiences. To give an example, a recent World Health Organization review on control measures for influenza cited no simulation studies but only real-world observational data [46].

Quarantine-related studies from real SARS outbreaks typically present the epidemic curve with case counts plotted against the timeline of events including arrival of index cases, and changes in public health response. In many reports [22,34,39,40], the impact of control measures is implied from qualitative observation, such as visible deceleration of new cases in plots, or the eventual end of the outbreak. Some of these reports appeared well after the outbreak, with efforts made to complete data on onset dates and transmission in hindsight (e.g., [42]). Drawing inference from epidemic curves suits only point-source outbreaks, and amount to one-group, pre-test post-test designs [47], providing weak evidence for causation. The true value of case reports, arguably, is the rich contextual information on challenges faced and unexpected events, and showing socio-political feasibility of aggressive control across settings [40].

Some case reports have taken a more quantitative approach. A few studies reported on the effect of quarantine in shortening the time from onset of symptoms to isolation [48], or the proportion of SARS cases who developed symptoms while already under quarantine [49]. Others report quarantine yield (the proportion of individuals quarantined who eventually develop SARS) [36], which reflects specificity of contact tracing versus burden from unnecessary quarantine. These are intermediate outcomes, however, evaluating processes as opposed to final outcomes.

In others, control interventions are examined pre- post-quarantine in relation to subsequent changes in R [4,43]. Wallinga and Teunis, 2004 [43], present the average daily effective reproductive number, R, both prior to alert and after, for each of four SARS outbreak locations. Table 1 of their analysis reveals fairly consistent transmission numbers pre-alert, but more variable R values post-alert, highlighting less effective control in Ontario relative to elsewhere. This is also an uncommon example of presentation of R estimates along with confidence limits from observed (as opposed to simulated) data. Their analysis would permit reporting of differences in transmissions before and after alert, but no reduction in R attributable to control was presented (with or without confidence limits). The approach also permitted no consideration of individual-level covariates.

### Quantitative estimates of quarantine impact

We estimated that use of community quarantine in the 2003 Ontario SARS outbreak reduced transmission to one third, with an absolute difference of 0.13 secondary cases per index case under quarantine, relative to not quarantined by symptom onset. For discussion purposes, we present several effect measures, including Secondary Case Count Difference (SCCD) and "number needed to quarantine" (NNQ), a novel adaptation of NNT. Our point estimate of NNQ for the Ontario outbreak was 7.5 persons in quarantine to one SARS case averted using data from probable or suspected SARS cases. As a point estimate, this NNQ compares very favourably with NNTs reported for public health interventions such as chemoprophylaxis for leprosy [50] and meningococcal disease [51], or vaccination against pertussis [52] and influenza and pneumococcal disease [53], particularly with a condition like SARS with significant morbidity and a high case-fatality rate. All estimates we present for the impact of quarantine, however, are imprecise. Bootstrapped confidence intervals include values for no impact. Statistical power is a limitation to this and many analyses of real outbreak data.

We also show, not surprisingly for an infection now known to be transmitted by droplet spread, a statistically significant association between the number of close contacts and number of secondary cases, per index case. Number of close contacts (level 1 in Table 1) had some overlap with the observed (non-significant) effect of quarantine, whereas the number of more distant contacts was unrelated to any apparent benefit of quarantine. Our analysis also suggests (without statistical significance) that reduction in the number of close contacts contributed to reduction in spread, and this may have implications for targeting of quarantine toward closer contacts [23,36].

### Statistical challenges

Research in quarantine effectiveness presents many challenges. One complication is the unit of analysis for the outcome relative to the intervention. Clinical decision-making looks at outcomes in the same individuals assigned to treatment. Interventions such as vaccination are more complex in that outcomes may be assessed at the individual or population level, with different implications, although the individual vaccinated is part of the same population. Number Needed to Vaccinate (NNV) has been estimated incorporating herd immunity [52]. The case of quarantine is distinct even from vaccination, in that all potential benefit is to other persons. It is theoretically possible to study sets of index cases and their contacts as independent units of analysis, although it is often difficult to identify precisely which persons exposed which others [43]. Here, we have worked with contacts matched to an exclusive index case [23]. The creation of sets of cases and associated controls goes only part-way toward a complete network-based analysis [33], although future studies could address non-independence of networks.

A second challenge to evaluators might be non-familiarity with regression models for count data. The generalized linear model used here to obtain an NNQ estimate, with a Poisson error term and identity link function, is less commonly used, but long-described in biostatistics texts. Regression models used here are available in all major statistical packages (Stata, SAS, SPSS and others). The distribution of secondary cases (per index case) may be positively skewed (with a few cases generating large numbers of transmissions [4]). Over-dispersion may need to be addressed through means such as use of negative binomial models in place of Poisson models, as above. Negative binomial models have interpretation very similar to Poisson models [26].

Statistical power was a limitation of our analysis and most studies of real outbreaks [54]. As the goal of outbreak management is to minimize events, small samples must be considered. Procedures assuming large samples tend to overstate precision relative to bootstrapping. Other authors in this field have used bootstrap variance methods as well [42].

### Methodological challenges

Random assignment of individuals to quarantine is not ethical; and in some jurisdictions, comprehensive quarantine procedures may eliminate any control arm [4]. In North America and Europe, voluntary quarantine practices are favoured and some degree of non-adherence is inevitable [6,22], so both non-quarantined and quarantined groups will be observed. However, selection bias related to health status, employment, family structure and other factors may confound the association between quarantine status and observed transmissions. The best possible observational design would permit evaluation of the decision made by public health officials to place individuals under quarantine and apply analyses based on both the intention to treat approach and taking compliance into account.

Retrospectively, we explored the possibility of identifying all individuals screened by public health staff for potential quarantine and contact tracing, regardless of final disposition. This was not feasible. Practices varied with respect to when a record was initiated (i.e., in one health unit a file might have been opened even with an unfounded inquiry, elsewhere a record was generated only with a confirmed contact link and symptoms). Within Public Health records, we were able to confirm 140 quarantined false positive "cases". These cases were quarantined contacts who became ill with possible SARS symptoms but were subsequently excluded as SARS cases, and they had at least one identified community contact. Future cost-benefit studies should include information on such groups (e.g., [36]). Legitimate costs are incurred for and by these false positive cases and their contacts which should be taken into account. Because people without the disease can't spread it, uneven distribution of such individuals across infection control conditions being compared could bias estimates of impact. As we found, the rate of false positive cases (resulting in no transmission) may have a large influence on the apparent benefit. Several case-reports discussed problems with prospective record-keeping, in terms of detailed contact tracing and the implications of time-lags in serological testing (including those never tested), as challenges to both outbreak management and research [4,35,41].

Measurement error is also likely with other important information, such as documenting level of contact and therefore numbers of individuals at risk by contact level. With delayed contact tracing, assignment of contact level may even be done after secondary infection (unblinded) and so could be biased toward closer contact where transmission already happen, and toward less close contact where the contact remained well.

Papers on the SARS experience have spoken about the importance of data management resources and described core data to be tracked during an outbreak (e.g., [55]). None made explicit recommendations for statistical evaluation of control measures. Planning to report statistics familiar to other areas of health care evaluation may improve the quality and comparability of data collected.

Our approach demonstrates that existing outbreak data may yield more information to evaluate outbreak control measures than has been reported. Further research, presenting quantitative differences in outcomes attributable to measures such as quarantine, would be useful in many ways. First, this would add to evidence on cost-effectiveness. Second, it would facilitate further methodological development in this field. Pooled re-analysis of existing outbreak data across several settings, would ameliorate statistical power problems, and increase the scientific contribution from these important databases.

### Challenges in interpretation and communication

Policy-makers rely on estimates of the impact of population-based preventive measures, which should derive from actual experience, as well as theoretical forecasting. Evidence also needs to be understood. It has been debated whether the NNT statistic achieves its goal to facilitate decision-making in other health care settings [17-20]. Further thought and discussion are needed as to how meaningful a NNQ statistic might be for decision-making in outbreak planning, relative to other expressions of attributable case reductions, such as SCCD also proposed here, or other metrics.

No variant on attributable risk difference or NNT can be interpreted without consideration of the absolute costs of not acting, and the harm side of the decision [56]. This discussion must include the severity of the illness, as well as harms of intervention to the individual and society which are difficult to quantify and value-laden [6]. Quarantine includes potential non-health related harms including civil liberties and may include economic and other costs to the individual [57].

Finally, studies to evaluate control measures for one agent may not be generalizable to other agents. Measures to restrict close contact probably made an important contribution to the control of SARS outbreaks [46]. Evidence of this is accumulating slowly and should be taken into consideration for future outbreaks of SARS or similar droplet spread agents without significant transmission in the asymptomatic phase. However, the applicability of this evidence to the current experience with pandemic influenza is less certain. Ferguson et al [58] present simulation data suggesting community quarantine and isolation may play a role in influenza control but comment that this would presume such measures were feasible. Transmission patterns for influenza however, make it less likely that contact tracing and quarantine would be fast enough to avoid transmission which is greatest in the earliest stage of infection [46]. Under such circumstances, use of a 'severe' [57] measure such as quarantine is likely not justified where such efforts are likely to yield little benefit.

## Conclusions

Relative to other health policy areas, literature on quarantine tends to lack in quantitative expressions of effectiveness, or agreement on how best to report differences in outcomes attributable to control measure. The study of quarantine effectiveness presents several methodological and statistical challenges. Further research and discussion are needed to understand the costs and benefits of enacting quarantine, and this includes a discussion of how quantitative benefit should be communicated to decision-makers and the public, and evaluated.

## Competing interests

There are no financial or non-financial competing interests. The funding source had no role in study design; collection, analysis or interpretation of data; writing of the report; nor decision to submit the paper for publication.

## Authors' contributions

All listed authors made substantial contributions to conception and design of the analysis presented in this manuscript. SB, ER and JL were involved in initial collection of data for the underlying outbreak database. All authors were involved in defining the objectives for the present analysis; in determining case selection and definitions for the present analysis; and in interpretation of results. SB and JL took lead responsibility for data analysis. All authors were involved drafting the manuscript and revising it critically for intellectual content. All authors have given final approval of the version to be published.

## Pre-publication history

The pre-publication history for this paper can be accessed here:

http://www.biomedcentral.com/1471-2458/9/488/prepub

## References

[B1] Heymann D Davis JR, Lederberg J Introduction Emerging Infectious Disease from the Global to the Local Perspective 2001 Washington, DC: Institute of Medicine. National Academy Press 29 34

[B2] Barbera J Macintyre A Gostin L Inglesby T O'Toole T DeAtley C Tonat K Layton M Large-Scale Quarantine Following Biological Terrorism in the United States: Scientific Examination, Logistic and Legal Limits, and Possible Consequences JAMA 2001 286 2711 2717 10.1001/jama.286.21.2711 11730447

[B3] Singer PA Benatar SR Bernstein M Daar AS Dickens BM MacRae SK Upshur REG Wright L Shaul RZ Ethics and SARS: lessons from Toronto BMJ 2003 327 1342 1344 10.1136/bmj.327.7427.1342 14656848 PMC286332

[B4] Anderson RM Fraser C Ghani AC Donnelly CA Riley S Ferguson NM Leung GM Lam TH Hedley AJ Epidemiology, Transmission Dynamics and Control of SARS: The 2002-2003 Epidemic Philos Trans R Soc Lond B Biol Sci 2004 359 1447 1091 1105 15306395 10.1098/rstb.2004.1490 PMC1693389

[B5] Reich DS Modernizing local responses to public health emergencies: Bioterrorism, epidemics, and the Model State Emergency Health Powers Act Journal of Contemporary Health Law and Policy 2003 19 379 414 14748251

[B6] Lemon SM Hamburg MA Sparling PF Choffnes ER Mack A Ethical and legal considerations in mitigating pandemic disease: workshop summary 2007 Washington, DC: Institute of Medicine. National Academy of Sciences 21595110

[B7] Schabas R Severe acute respiratory syndrome: Did quarantine help? Can J Infect Dis Med Microbiol 2004 15 4 204 18159492 10.1155/2004/521892 PMC2094974

[B8] Knobler S Mahmoud A Lemon S Pray L Eds The Global Application of Knowledge, Tools, and Technology: Opportunities and Obstacles The Impact of Globalization on Infectious Disease Emergence and Control: Exploring the Consequences and Opportunities Workshop Report 2006 Washington, DC: Institute of Medicine, National Academy of Sciences 80 124

[B9] Bauch CT Lloyd-Smith JO Coffee MP Galvani AP Dynamically Modeling SARS and Other Newly Emerging Respiratory Illnesses: Past, Present and Future Epidemiology 2005 16 791 801 10.1097/01.ede.0000181633.80269.4c 16222170

[B10] Bensimon CM Upshur RE Evidence and effectiveness in decision making for quarantine American Journal of Public Health 2007 97 S44 S48 10.2105/AJPH.2005.077305 17413076 PMC1854977

[B11] Egger M Davey G Smith Altman DG Systematic Reviews in Health Care: Meta-analysis in context 2001 2 London: BMJ Books

[B12] Petitti DB Meta-Analysis, Decision Analysis, and Cost-Effectiveness Analysis: Methods for Quantitative Synthesis in Medicine 2000 Oxford: Oxford University Press

[B13] Gray JAM Egger M, Davey Smith G, Altman DG Using systematic reviews for evidence based policy making Systematic Reviews in Health Care: Meta-Analysis in Context 2000 2 Oxford: BMJ Books 410 418

[B14] Gray JAM Evidence-based healthcare: how to make health policy and management decisions 1997 London: Churchill Livingstone

[B15] Greenland S Rothman JK Lash TL Rothman JK, Greenland S, Lash TL Measures of Effect and Measures of Association Modern Epidemiology 2008 3 Philadelphia: Lippincott Williams & Wilkins 51 70

[B16] Altman DG Schulz KF Moher D Egger M Davidoff F Elbourne DR Gøtzsche PC Lang T for the CONSORT group The revised CONSORT statement for reporting randomized trials: explanation and elaboration Annals of Internal Medicine 2001 134 663 694 11304107 10.7326/0003-4819-134-8-200104170-00012

[B17] Bender R Armitage P, Colton T Number Needed to Treat (NNT) Encyclopedia of Biostatistics 2009 2 Chichester: Wiley

[B18] Cook RJ Sackett DK The number needed to treat: a clinically useful measure of treatment effect BMJ 1995 310 452 454 7873954 10.1136/bmj.310.6977.452 PMC2548824

[B19] McAlistser FA The "number needed to treat" turns 20 - and continues to be used and misused Canadian Medical Association Journal 2008 179 549 553 10.1503/cmaj.080484 18779528 PMC2527399

[B20] Freemantle N Mason JM Eccles M Deriving treatment recommendations from evidence within randomised trials: the role and limitation of meta analysis International Journal of Technology Assessment in Health Care 1999 15 304 315 10507190

[B21] Anderson RM May RM Infectious diseases of humans: dynamics and control 1991 Oxford: Oxford University Press

[B22] Svoboda T Henry B Shulman L Kennedy E Rea E Ng W Wallington T Yaffe B Gournis E Vicencio E Public Health Measures to Control the Spread of the Severe Acute Respiratory Syndrome during the Outbreak in Toronto The New England Journal of Medicine 2004 350 2352 2361 10.1056/NEJMoa032111 15175437

[B23] Rea E Laflèche J Stalker S Guarda BK Shapiro H Johnson I Bondy SJ Upshur R Russell ML Eliasziw M Duration and distance of exposure are important predictors of transmission among community contacts of Ontario SARS cases Epidemiology and Infection 2007 135 914 921 10.1017/S0950268806007771 17217552 PMC2870656

[B24] Rogers WH Regression standard errors in clustered samples Stata Technical Bulletin 1993 13 19 23

[B25] StataCorp Stata Statistical Software: Release 10 (SE) 2007 College Station, TX: StataCorp LP

[B26] Agresti A Categorical Data Analysis 2002 2 New Jersey: John Wiley & Sons

[B27] Seeber GUH Armitage P, Colton T Poisson Regression Encyclopedia of Biostatistics 2009 2 Chichester: Wiley

[B28] North D Number needed to treat BMJ 1995 310 1269 7767217 10.1136/bmj.310.6989.1269 PMC2549644

[B29] Shrout PE Bolger N Mediation in experimental and nonexperimental studies: new procedures and recommendations Psychological Methods 2002 7 422 445 10.1037/1082-989X.7.4.422 12530702

[B30] Efron B Tibshirani RJ An Introduction to the Bootstrap 1993 New York: Chapman & Hall

[B31] Baron RM Kenny DA The moderator-mediator variable distinction in social psychological research: conceptual, strategic, and statistical considerations J Pers Soc Psychol 1986 51 6 1173 1182 3806354 10.1037/0022-3514.51.6.1173

[B32] Hsieh YH King CC Chen CWS Ho MS Hsu SB Wu YC Impact of quarantine on the 2003 SARS outbreak: A retrospective modeling study Journal of Theoretical Biology 2007 244 729 736 10.1016/j.jtbi.2006.09.015 17055533 PMC7094157

[B33] Epstein JM Lemon SM, Hamburg MA, Sparling PF, Choffnes ER, Mack A Remarks on the role of modeling in infectious disease mitigation and containment Ethical and legal considerations in mitigating pandemic disease: workshop summary 2007 Washington, DC: Institute of Medicine. National Academy of Sciences 105 112

[B34] Bell DM World Health Working Group on International and Community Transmission of SARS Public health interventions and SARS spread, 2003 Emerging Infectious Diseases 2004 10 1900 1906 15550198 10.3201/eid1011.040729 PMC3329045

[B35] Chau PH Yip PSF Monitoring the severe acute respiratory syndrome epidemic and assessing effectiveness of interventions in Hong Kong Special Administrative Region Journal of Epidemiology and Community Health 2003 57 766 769 10.1136/jech.57.10.766 14573569 PMC1732291

[B36] Ooi PL Lim S Chew SK Use of quarantine in the control of SARS in Singapore American Journal of Infection Control 2005 33 252 257 10.1016/j.ajic.2004.08.007 15947741 PMC7119078

[B37] Ou J Li Q Zeng G Dun Z Qin A Efficiency of quarantine during an epidemic of Severe Acute Respiratory Syndrome--Beijing, China, 2003 Morbidity and Mortality Weekly Report 2003 52 1037 14586295

[B38] Pang X Zhu Z Xu F Guo J Gong X Liu D Liu Z Chin DP Feikin DR Evaluation of Control Measures Implemented in the Severe Acute Respiratory Syndrome Outbreak in Beijing, 2003 JAMA: The Journal of the American Medical Association 2003 290 3215 3221 10.1001/jama.290.24.3215 14693874

[B39] Riley S Fraser C Donnelly CA Ghani AC Abu-Raddad LJ Hedley AJ Leung GM Ho LM Lam TH Thach TQ Transmission Dynamics of the Etiological Agent of SARS in Hong Kong: Impact of Public Health Interventions Science 2003 300 1961 1966 10.1126/science.1086478 12766206

[B40] S.A.R.S. Investigation Team from D.M.E.R.I. and S. G. H. Strategies adopted and lessons learnt during the severe acute respiratory syndrome crisis in Singapore Reviews in Medical Virology 2005 15 57 70 10.1002/rmv.458 15565739

[B41] Tuan PA Horby P Dinh PN Mai LTQ Zambon M Shah J Huy VQ Bloom S Gopal R Comer J Plant A SARS transmission in Vietnam outside of the health-care setting Epidemiology and Infection 2007 135 392 401 10.1017/S0950268806006996 16870029 PMC2870589

[B42] Yip P-S Hsieh YH Xu Y Lam KF King CC Chang HL Assessment of Intervention Measures for the 2003 SARS Epidemic in Taiwan by Use of a Back Projection Method Infection Control and Hospital Epidemiology 2007 28 525 530 10.1086/516656 17464910

[B43] Wallinga J Teunis P Different Epidemic Curves for Severe Acute Respiratory Syndrome Reveal Similar Impacts of Control Measures American Journal of Epidemiology 2004 160 509 516 10.1093/aje/kwh255 15353409 PMC7110200

[B44] Castillo-Chavez C Castillo-Garsow CW Yakubu AA Mathematical Models of Isolation and Quarantine JAMA: The Journal of the American Medical Association 2003 290 2876 2877 10.1001/jama.290.21.2876 14657077

[B45] Timpka T Ericksson H Gursky EA Nyce JM Morin M Jenvald J Stromgren M Holm E Ekberg J Population - based simulations of influenza pandemics: validity and significance for public health policy Bulletin of the World Health Organization 2009 87 305 311 10.2471/BLT.07.050203 19551239 PMC2672572

[B46] World Health Organization Writing Group Nonpharmaceutical interventions for pandemic influenza, international measures Emerging Infectious Diseases 2006 12 81 87 16494722 10.3201/eid1201.051370 PMC3291414

[B47] Cook TD Campbell DT Quasi-Experimentation: Designs & Analysis Issues for Field Settings 1979 Boston: Houghton Mifflin Company

[B48] Hsieh YH King CC Chen CW Ho MS Lee YJ Liu FC Wu YC Julian JS Wu Quarantine for SARS, Taiwan Emerging Infectious Diseases 2005 11 278 282 15752447 10.3201/eid1102.040190 PMC3320446

[B49] Wang TH Wei KC Hsiung CA Maloney SA Eidex RB Posey DL Chou WH Shih WY Kuo HS Optimizing severe acute respiratory syndrome response strategies: lessons learned from quarantine American Journal of Public Health 2007 97 S98 S100 10.2105/AJPH.2005.082115 17413071 PMC1855001

[B50] Moet FJ Pahan D Oskam L Richardus JH CSG Effectiveness of single dose rifampicin in preventing leprosy in close contacts of patients with newly diagnosed leprosy: cluster randomised controlled trial BMJ 2008 336 761 764 10.1136/bmj.39500.885752.BE 18332051 PMC2287265

[B51] Purcell B Samuelsson S Hahne SJM Ehrhard I Heuberger S Camaroni I Charlett A Stuart JM Effectiveness of antibiotics in preventing meningococcal disease after a case: systematic review BMJ 2004 328 1339 10.1136/bmj.328.7452.1339 15178612 PMC420283

[B52] Van Rie A Hethcote HW Adolescent and adult pertussis vaccination: computer simulations of five new strategies Vaccine 2004 22 3154 3165 10.1016/j.vaccine.2004.01.067 15297068

[B53] Kelly H Attia J Andrews R Heller RF The number needed to vaccinate (NNV) and population extensions of the NNV: comparison of influenza and pneumococcal vaccine programmes for people aged 65 years and over Vaccine 2004 22 2192 2198 10.1016/j.vaccine.2003.11.052 15149776

[B54] Cai QC Xu QF Xu JM Guo Q Cheng X Zhao GM Sun QW Lu J Jiang QW Refined Estimate of the Incubation Period of Severe Acute Respiratory Syndrome and Related Influencing Factors American Journal of Epidemiology 2006 163 211 216 10.1093/aje/kwj034 16339050 PMC7109871

[B55] Scott RD Gregg E Meltzer MI Collecting data to assess SARS interventions.(Dispatches) Emerging Infectious Diseases 2004 10 1290 15324551 10.3201/eid1007.030749 PMC3323351

[B56] Sinclair JC Cook RJ Guyatt GH Pauker SG Cook DJ When should an effective treatment be used? Derivation of the threshold number needed to treat and the minimum event rate for treatment J Clin Epidemiol 2001 54 253 262 10.1016/S0895-4356(01)00347-X 11223323

[B57] Gostin L Public Health Strategies for Pandemic Influenza: Ethics and the Law JAMA 2006 295 1700 1704 10.1001/jama.295.14.1700 16609092

[B58] Ferguson NM Cummings DAT Fraser C Cajka JC Cooley PC Burke DS Strategies for mitigating an influenza pandemic Nature 2006 442 448 452 10.1038/nature04795 16642006 PMC7095311

